# A Model Integration Pipeline for the Improvement of Human Genome-Scale Metabolic Reconstructions

**DOI:** 10.1515/jib-2018-0068

**Published:** 2018-12-21

**Authors:** Vítor Vieira, Jorge Ferreira, Rúben Rodrigues, Filipe Liu, Miguel Rocha

**Affiliations:** Center of Biological Engineering, University of Minho – Campus de Gualtar, Braga, Portugal; Argonne National Laboratory, Lemont, IL, USA

**Keywords:** Genome-scale metabolic models, human metabolism, omics databases, database integration

## Abstract

Metabolism has been a major field of study in the last years, mainly due to its importance in understanding cell physiology and certain disease phenotypes due to its deregulation. Genome-scale metabolic models (GSMMs) have been established as important tools to help achieve a better understanding of human metabolism. Towards this aim, advances in systems biology and bioinformatics have allowed the reconstruction of several human GSMMs, although some limitations and challenges remain, such as the lack of external identifiers for both metabolites and reactions. A pipeline was developed to integrate multiple GSMMs, starting by retrieving information from the main human GSMMs and evaluating the presence of external database identifiers and annotations for both metabolites and reactions. Information from metabolites was included into a graph database with omics data repositories, allowing clustering of metabolites through their similarity regarding database cross-referencing. Metabolite annotation of several older GSMMs was enriched, allowing the identification and integration of common entities. Using this information, as well as other metrics, we successfully integrated reactions from these models. These methods can be leveraged towards the creation of a unified consensus model of human metabolism.

## 1 Introduction

The recent developments in the “omics” fields and related technologies, together with advanced computational tools, helped the scientific community understand the different layers of human cells’ genotypes and phenotypes, increasing our knowledge of how certain changes lead to disease, especially when looked at a genome-scale level [1]. Metabolism can be seen as the main system to ensure adequate regulation of human cells, since it is responsible for the connections between the genetic content of a human cell and external environmental factors, consisting of a set of interconnected biochemical reactions [2].

Since the available amount of omics data is increasing in a rapid way, computational tools have been an important asset to process all the gathered information. Genome-scale Metabolic Models (GSMMs) are an example of such tools, providing mathematical representations of molecular entities at a system level that can help grasp the connection between genome and metabolism [3], [4].

The process to obtain a model representing a set of biochemical transformations that may occur in the cell, begins with a Genome-scale reconstruction (GENRE), which can also be represented as a metabolic network. Then, there is the need to curate the functional genome annotation through literature review and manual curation using available experimental data. The four main four steps for the model reconstruction are, thus, the draft reconstruction, model curation, creation and validation.

**Figure 1:**
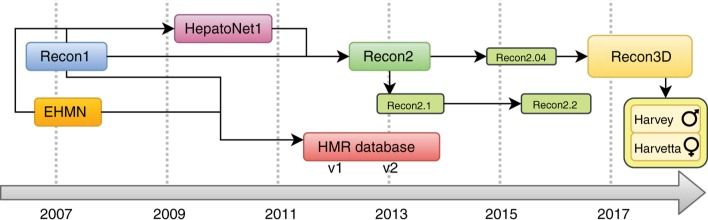
Timeline of the development of human GSMMs from 2007 until the present day. This accounts only for generic models but the HepatoNet1 hepatocyte model is included as a contribution for Recon2. Each model is represented by a rounded box. Revisions and derivative models are distinguished by a black border and smaller font.

Throughout the last 10 years, several models of the human cell metabolism have been developed (Figure 1). The first GSMMs were mainly focused on central carbon metabolism and even before the development of the first human GSMMs, there were also representations of the human mitochondria [5] and fibroblast [6]. Although smaller in scale, these models led the scientific community to acquire better insights on cell metabolism.

With human genome sequencing as a starting point, both Recon1 [7] and the Edinburgh human metabolic network (EHMN) [8] were developed in 2007. Since both models tried to encompass the whole human metabolism, complementary efforts were made to create tissue-specific metabolic models (based on the existing models) for hepatocyte cells, the HepatoNet1 [9].

A few years later, a new version of the Recon model was presented by Thiele et al. [10], in parallel with another model, represented as the Human Model Reaction (HMR) database [4]. With the growing knowledge on human metabolism, the Recon2 model (comprising parts of the metabolism represented in the HepatoNet1 and EHMN) was subjected to various updates/revisions. These include improved gene-protein-reaction rules according to new discoveries of the human genome [11] (with a web visualization tool to help navigate the metabolic model), while others adjusted pathways to provide more reliable predictive capabilities [12] and even improved chemical balancing [13].

The most recent human metabolic model is Recon3D [14]. This model is an improvement on the previous version (around 6000 extra reactions) which also incorporates a large part of the second version of the HMR model (approximately 2500 reactions) [15]. This model was used for the first gender-specific whole-body metabolic models, Harvey (male) and Harvetta (female). Not only do the models consider most human tissue types, they also contain information on microbial communities present in the human body, allowing distinct types of analysis [16].

Different human models and other sources may be merged into a consensus metabolic model, integrating relevant biological entities (metabolites and reactions) shared between two or more models based on their information and external databases. However, those entities are typically identified without a shared standard, which is a possible limitation for the integration process.

Metabolites present in human models can typically include information such as their name, InChI key, chemical formula, identifiers for external databases, such as HMDB [17], KEGG [18], PubChem [19], CHEBI [20], LipidMaps [21], DrugBank [22] or BRENDA [23] storing metabolite and reaction information, and model identifiers that can be used to connect identical components of different models. As an example, if a model *A* contains a metabolite *M*1 that has an external database identifier also found on a metabolite *C*1 of model *B*, both *M*1 and *C*1 can be considered identical, a property that can be leveraged to integrate the two models. Building over the incremental identification of shared metabolites, we can also infer shared reactions (and pathways) present in the models.

In this work, we will apply a cross-reference integration process to find the shared metabolites across different models, and consequently increase the available information for the metabolites present in each model. Also, using the previously described property, we will perform an integration of reactions of the same models by using the processed metabolites to connect their reactions.

## 2 Workflow

The pipeline devised for this work includes three main steps, namely data retrieval and preprocessing, metabolite integration and subsequent integration of reactions.

### 2.1 Data Retrieval

For this work, we used most of the available human metabolic models. Table 1 shows an overview of the information used to perform this work, obtained from the Systems Biology Markup Language (SBML) files of the models, using the COBRApy package.

**Table 1:** Information for the human metabolic models used in this work.

	#Metabolites	ChEBI	DrugBank	HMDB	KEGG Compound	LipidMaps	PubChem Compound
Recon1	1245						
Recon1 (with drains)	1245						
HepatoNet1	712						
Edinburgh HMN	2715						
HMR (adipocyte)	6170	⚫			⚫	⚫	
HMR (generic)	9267						
HMR2.0**	3532	⚫		⚫	⚫		
iHuman2207	6341						
Recon2	2360	⚫		⚫	⚫		
Recon2 (HEK cell)	2288	⚫		⚫	⚫		⚫
Recon2.1	2360	⚫		⚫	⚫		
Recon2.2	2478	⚫	⚫	⚫	⚫		
Recon3D	4140	⚫		⚫	⚫		⚫
Recon3DModel	2797	⚫		⚫	⚫		⚫

The symbol ⚫ represents that at least one metabolite has information for that database. The symbol ** indicates that the extra information for this model was obtained from the .xls file on http://www.metabolicatlas.org/downloads/hmr.

**Figure 2:**
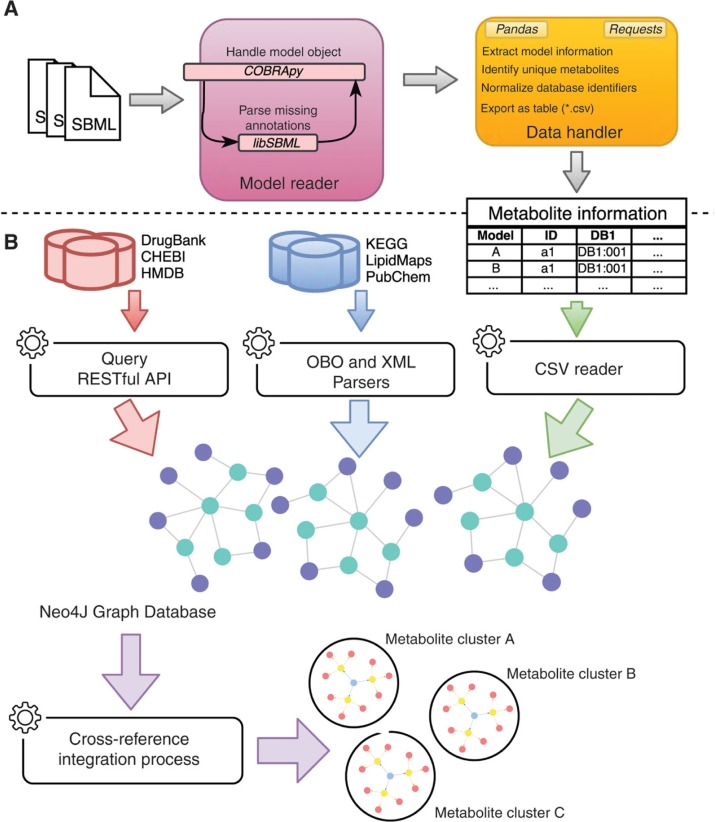
Representation of the data retrieval pipeline employed in this work. (A) Preprocessing metabolite information from the SBML files: the resulting table contains data for all metabolites of the used models. (B) Preprocessing metabolite information from external databases using Query RESTful API, OBO and XML parsers; Reading metabolite information from models; Insertion of metabolite information into Neo4J graph database; Identification of metabolite clusters using a cross-reference integration process: the result of these steps is a set of metabolite clusters, where each cluster groups unique metabolite identifiers or data from databases and models.

Figure 2(A) shows the workflow for processing the metabolite information of each model. Firstly, all of the SBML models are read with the COBRApy package. Since the annotation field from the metabolites was not being parsed, that information was obtained using the libSBML package. To integrate the metabolites, a matrix was created where each row represents a metabolite for a given model and each column represents information for the integration, i.e. identifiers for CHEBI, DrugBank, etc.

### 2.2 Metabolite Integration

**Figure 3:**
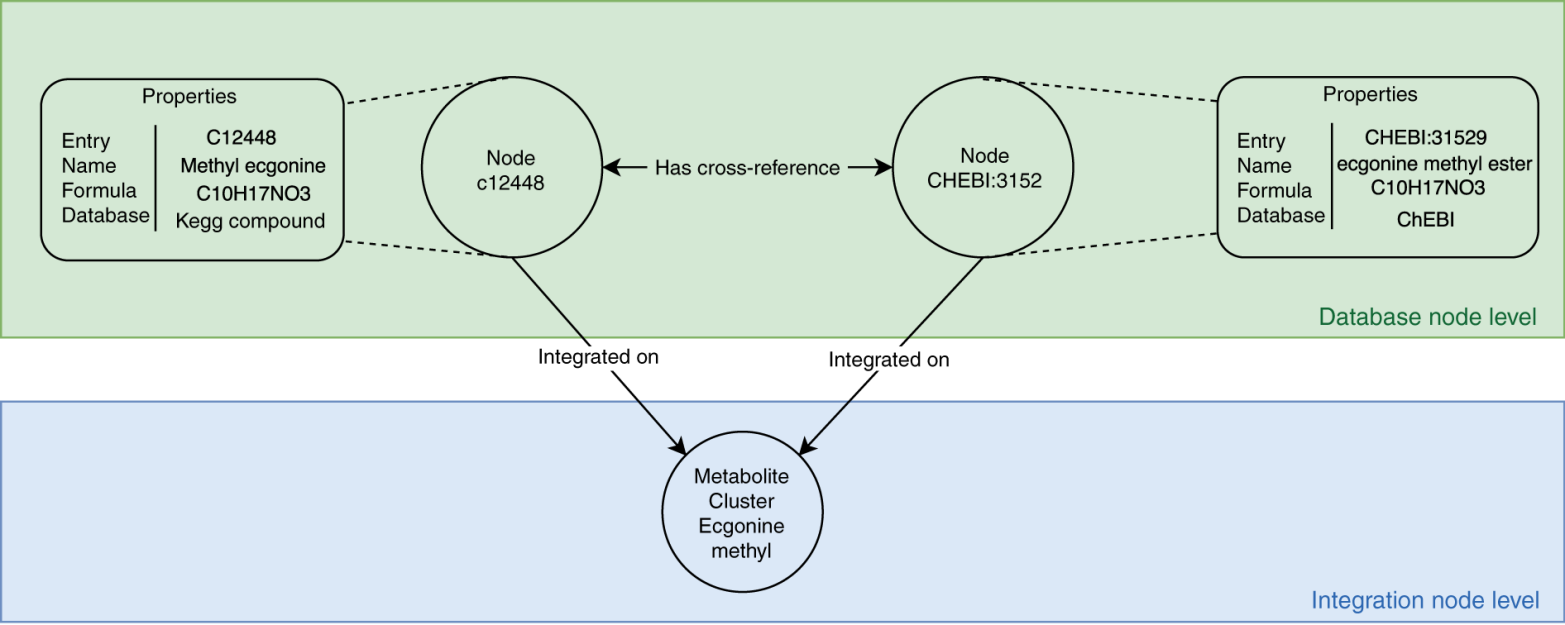
Data representation on the Neo4J database graph: the database is separated in two main levels: database and integration nodes. Database information is loaded into metabolite nodes that contain properties with metabolite information on the database node level; the integration process connects metabolite nodes representing the same metabolite information into cluster nodes that are present at the integration node level.

A metabolite integration tool was created using Java and a Neo4J graph database, which includes metabolite information from databases (CHEBI, DrugBank, HMDB, KEGG Compound, LipidMaps and PubChem Compound) and from human metabolic models (Figure 2B). Nodes on the graph database represent the metabolites from the databases and cross-references are represented by edges as shown in Figure 3.

The graph database was initially loaded with metabolite data from metabolic models containing references to external database identifiers. These were then used to query PubChem Compound, KEGG Compound and LipidMaps databases based on their RESTful application programming interfaces (APIs) using previously implemented loaders, while information from CHEBI, HMDB and DrugBank databases were loaded using OBO and XML parsers.

Identification of shared metabolites on different models was performed by a cross-reference integration process, which creates a second level of metabolite nodes on the graph database representing clusters of metabolite instances from the different databases. Each node has connections to metabolites from different models being referenced by a database identifier.

### 2.3 Reaction Integration

A process for reaction integration was devised, based on previously determined metabolite clusters. Firstly, we selected seven models for this process (Recon1, Recon2.04, Recon3D, HMR 1 and 2, HepatoNet and EHMN) since Recon3D integrates reactions from all of the other six models. In the next step, we exclude reactions that do not contain metabolites, since we would not be able to integrate them with the clusters. Also, reactions that involve more than one compartment are excluded to avoid integration of metabolic processes shifting metabolites across compartments and transport reactions involving a single metabolite in two compartments, since this information is not taken into account in the integration process, and also due to the fact that these reactions often do not represent enzymatic reactions (such as diffusion).

The metabolite clusters identified using the methods described in the previous section are then used to replace the original metabolites found in the metabolic models. Since a cluster *C* is a set of metabolites from different models that represent the same real metabolite, we assume each metabolite *m* ∈ *C* is now represented by its cluster *C*. Thus, each reaction will be represented by a set of pairs containing a metabolite cluster and a stoichiometric coefficient. This representation allows us to integrate reactions by finding those with identical clusters and stoichiometry for all involved metabolites in both the reactants and products of each reaction.

Our integration algorithm requires some important functions to be defined, namely *ρ*(*r*) yielding the set of cluster-stoichiometry pairs for a given reaction *r* and a *θ*(*m*) function returning the set of reactions in which the metabolite cluster *m* is involved. The process used for reaction integration is presented on Algorithm 1.

Algorithm 1 **Reaction integration algorithm employed in this work. At the beginning of each cycle, the first reaction (*R*_1_) of the set *R* is obtained, and every other reaction involving its clustered metabolites is stored in the set *P*. The intersection of all reaction sets in *P_i_* is then obtained and stored as the set *C*. All reactions *r* in *C* whose cluster-stoichiometry pairs are identical to *R*_1_ are added to the set *Y* of reaction clusters and removed from the set *R* so that the next iteration of the cycle will not group them.**

*Y* = {}

*R* = full set of reaction identifiers

**while** *R* ≠ {} **do**

   *P* = *θ*(*m*), ∀*m* ∈ *ρ*(*R*_1_)

   $C=\bigcap\limits_{i=1}^{|P|}P_{i}$

   $x=\{r,\ \forall r\in C:|\rho(R_{1})|=|\rho(r)|\}$

   *Y* = *Y* ∪ *x*

   *R* = *R* − *C*

**end**

The reaction clusters generated through this process are guaranteed to contain reactions with equal stoichiometry, assuming the metabolite clusters are correct. We further enhanced this clustering process by adding reactions to clusters if the external identifiers can be matched. This assumption can be considered valid on some cases where the model reconstruction includes reactions from previous models, as is the case of Recon 1 through 3 and HMR versions 1 and 2. This process is done iteratively through each pair of models deemed suitable for these comparisons.

The results from this work are available online on the following link: https://www.bio.di.uminho.pt/humanmodelintegration/

## 3 Application and Discussion

**Figure 4:**
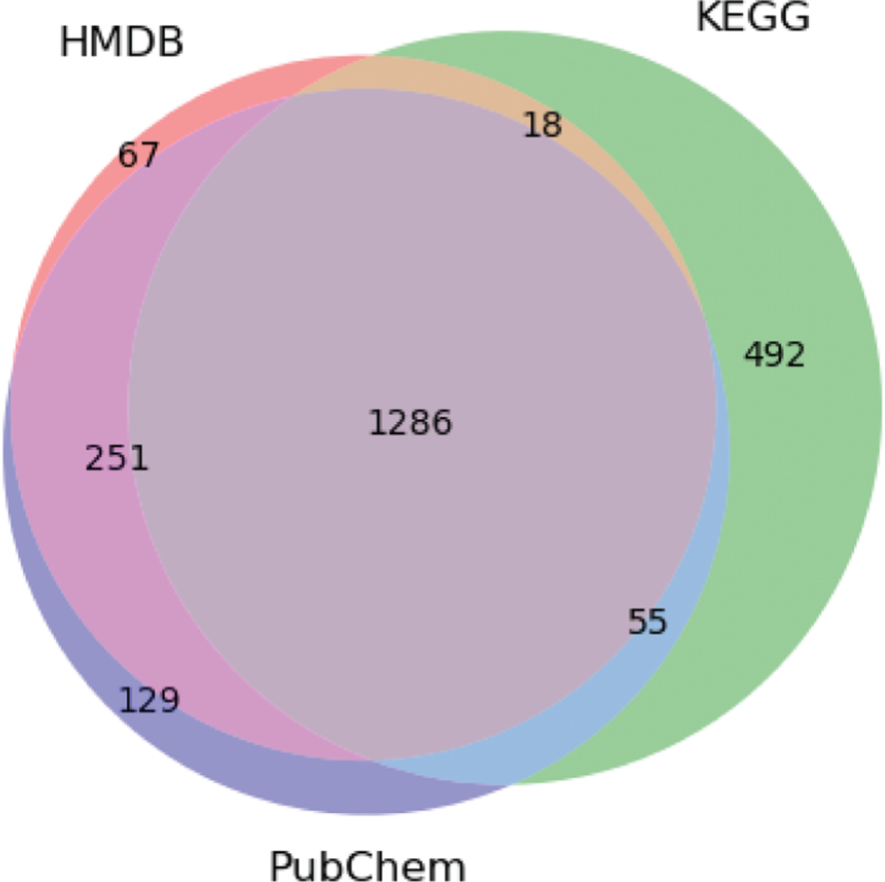
Venn diagram showing the integration of the HMDB, KEGG and PubChem Compound. The numbers represent the amount of metabolite clusters containing information from the three databases, as well as clusters with overlapping references.

**Figure 5:**
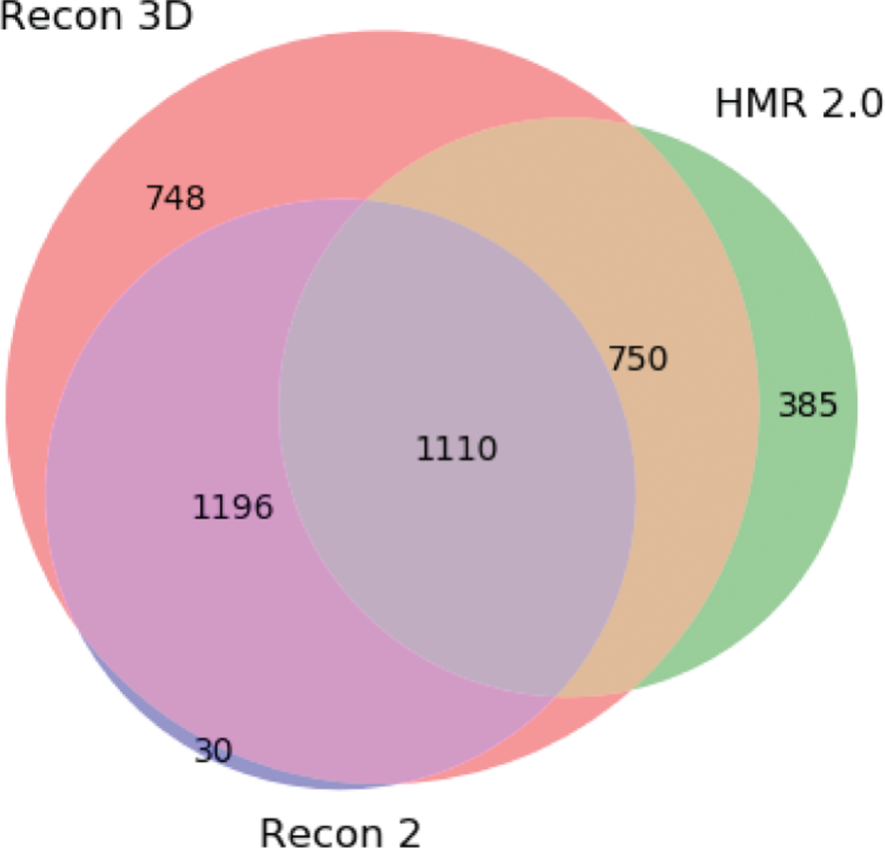
Venn diagram showing the integration of the Recon3D, Recon2 and HMR2.0. The numbers represent the amount of clusters with metabolites from each model, as well as clusters that integrate two or more models.

To assess the viability of the pipeline, first we show how the databases assist the integration/creation of the metabolite clusters (Figure 4 and Figure 5). In Figure 4, it is possible to see that KEGG has more information than HMDB and PubChem, reflecting the larger number of the clusters with only information for that database. In terms of integration, results were good, since most compounds of each database were matched with other databases; for instance, more than 80 % of KEGG compounds matched HMDB and PubChem databases.

Looking at Figure 5, we can clearly see that the new Recon model encompasses most of the clusters between the three models (90.1 % of the clusters). Although we do not know if some metabolites from the previous Recon version were removed from this new version, 30 clusters are not integrated with the newer one. However, we can see that the integration with HMR2.0 has improved over Recon2. An interesting fact is that Recon2 does not contain information about HMR2.0 and we were able to integrate at least 1110 metabolite clusters (around 50 %) from the latter model.

**Figure 6:**
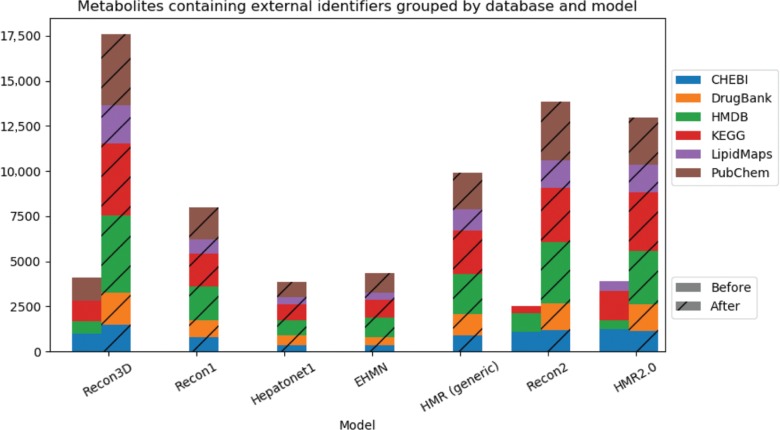
Cumulative bar charts representing the number of metabolites assigned with external identifiers for each database and model before (left bar) and after (right striped bar) the integration pipeline.

Figure 6 shows the evolution of the number of external identifiers for each model. Overall, the pipeline is able to significantly increase the number of metabolites which contain information to external databases. Even the most recent model was improved, with at least 400 new identifiers for its metabolites.

**Figure 7:**
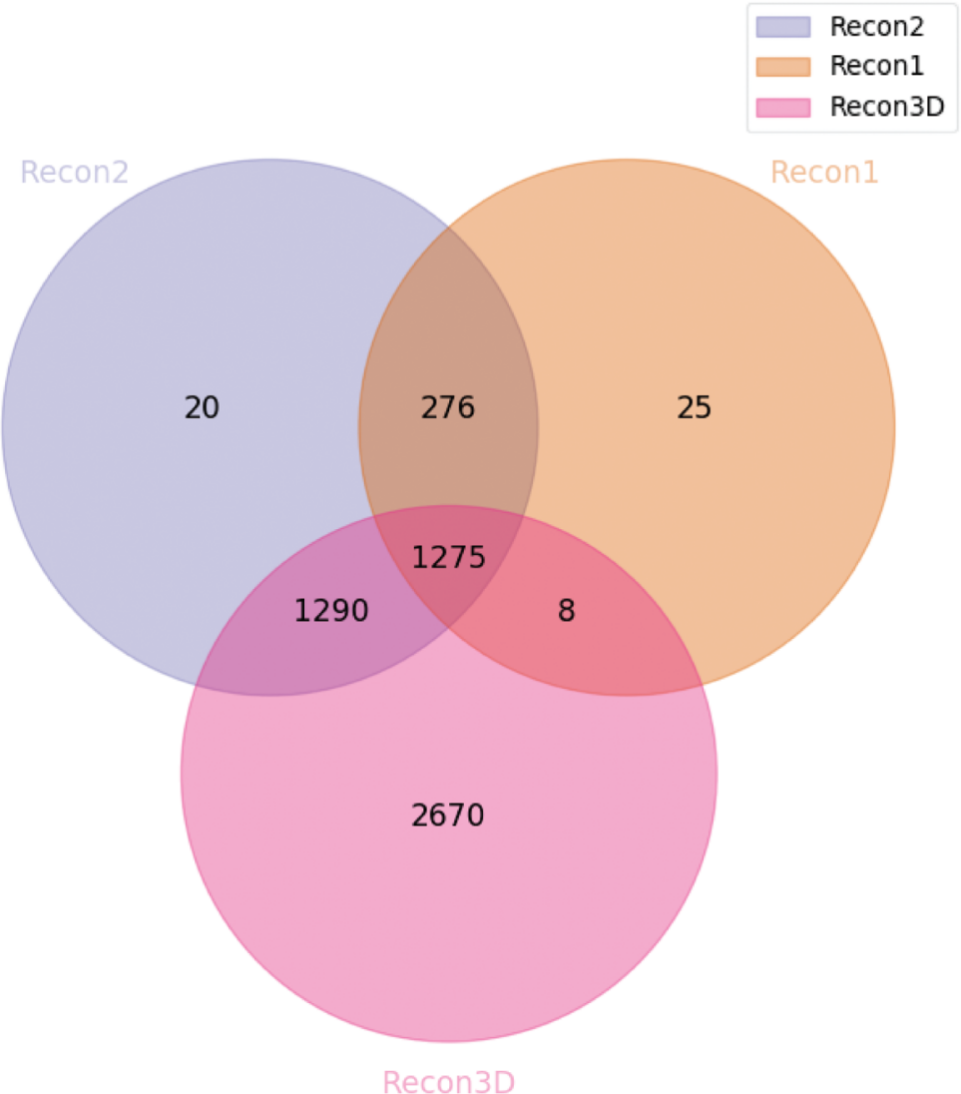
Venn diagram highlighting the number of reaction clusters integrated between the main three revisions of the Recon models.

When evaluating the effects of the integration of the reactions, we first decided to compare how the three Recon models have evolved. For that, we analysed the amount of intersected reaction clusters between these models (Figure 7).

We concluded that the majority of the Recon1 metabolites were integrated with the other two versions (with the exception of 25 clusters). For the Recon2, we reach a similar result. However, around 1300 clusters are only shared with Recon3D; those are most likely reactions from the models that were originally integrated with the Recon2 model coming from the HepatoNet and EHMN models. Regarding the Recon3D, about 2700 clusters were not integrated with the other models. Lastly, one unexpected result of our integration method is the fact that around 280 clusters are only shared by Recon1 and Recon2. When analyzing some of these clusters in Recon1 and 2, despite the stoichiometry being correct, we verified that the identifiers of the metabolites associated with some of these reactions do not match with the third version of the model. Therefore, it is likely that these metabolites could not be integrated using our tool.

**Figure 8:**
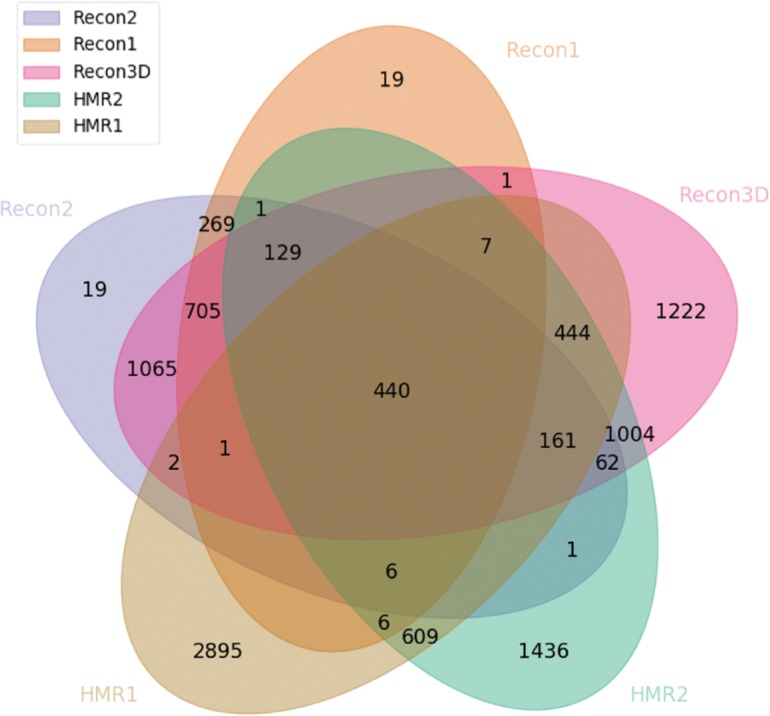
Venn diagram highlighting the number of reaction clusters integrated between all the main versions of Recon and HMR.

Since the last two versions of the Recon models were augmented with the other metabolic models, we performed an evaluation of our own integration of those models to assess how the models can be interconnected. We first decided to compare all the three versions of Recon with both HMR1 and HMR2.0 (Figure 8).

There are some relevant points to highlight from the Figure 8. First, it is noticeable that there is a large leap in terms of integration from Recon1 and 2 to Recon3. We also can see that a significant part of the HMR2 model is integrated with the Recon3D, which is expected due to the nature of the reconstruction of the latter. However, HMR1 is only integrated with the Recon models if they are associated with HMR2. There are 440 clusters which are integrated with all the three models. Since the revisions of the models do not include all of the information from their previous versions, the shared clusters are associated with a critical part of the metabolism of the human cell. It is clear that several efforts have been made to be able to integrate all the information of the analyzed models, although there is a great number of clusters of reactions that were not integrated.

**Figure 9:**
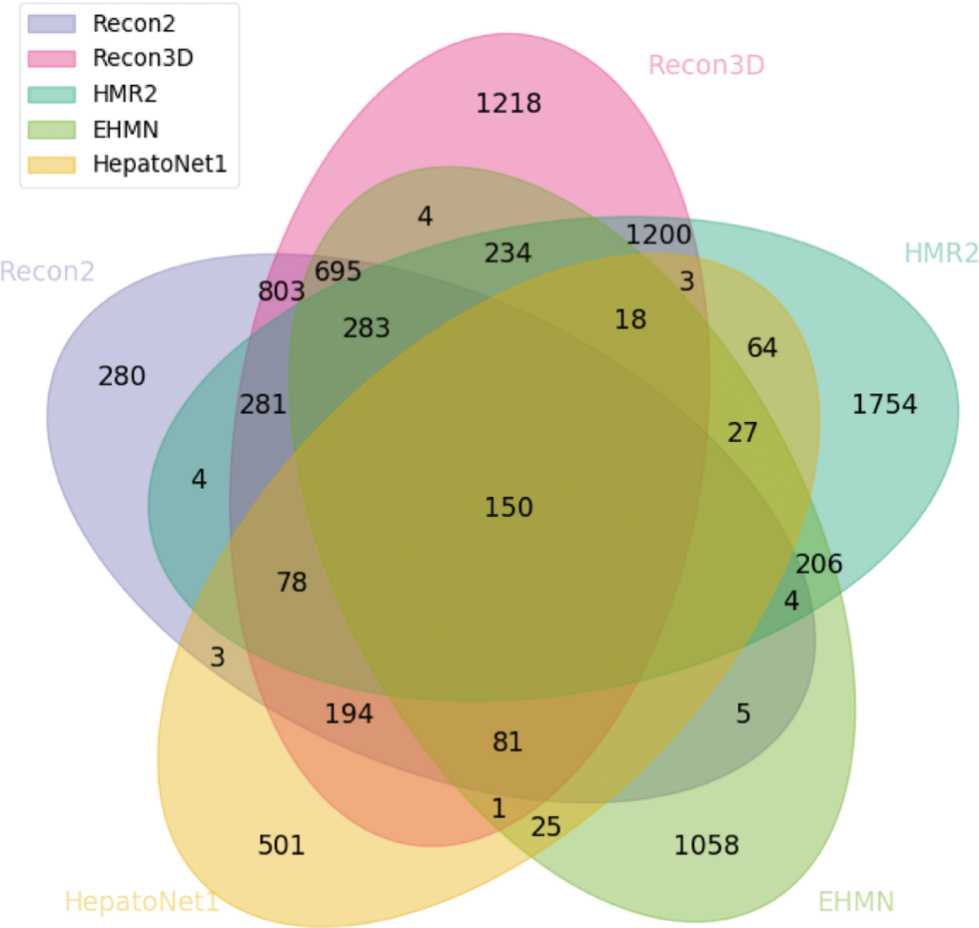
Venn diagram highlighting the number of reaction clusters integrated between the integrated models in both Recon2 and Recon3D.

Aside from the HMR models, EHMN and HepatoNet have also been integrated with the Recon models since its second iteration. We also verified this fact in our integration results (Figure 9). In this analysis, the Recon1 model was excluded since it has not been integrated with any model and had a smaller impact when compared with other models (Figure 8).

There is a sharp increase in the amount of unique clusters. Although Recon3D has a small decrease on this number, both HMR2 and Recon2 have increased theirs, probably due to the absence of HMR1 and Recon1, respectively. Looking at the clusters all five models share, the number is lower than in the previous analysis (Figure 8), since HepatoNet is a context-specific model for hepatocytes which naturally have less common reactions.

**Figure 10:**
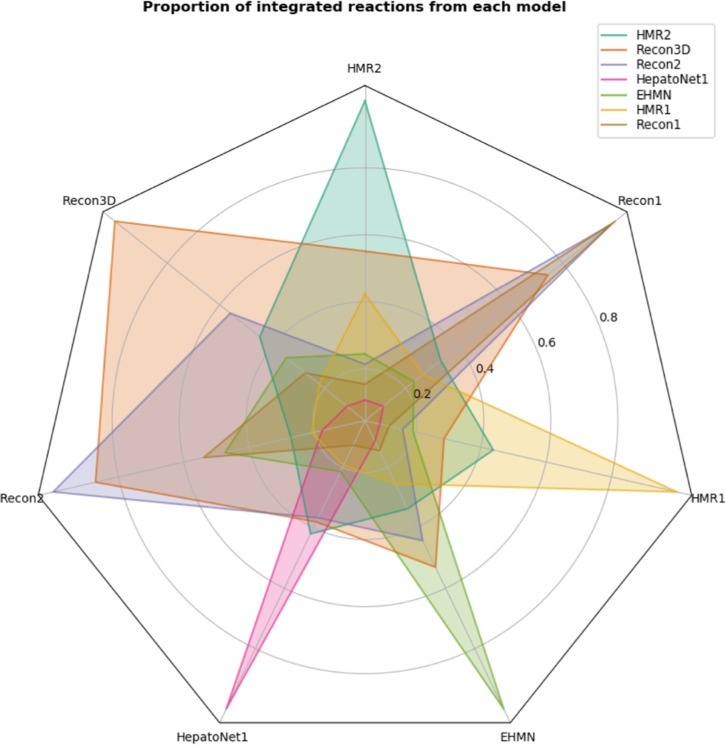
Radar plot representing the proportion of reactions that each model contains from the others. Each coloured polygon represents a single model and each of its vertices represent the proportion of reactions shared by other models. This proportion is always 1 in the vertex associated with its own model. Recon3D, as expected, is the model that contains more information from all the other models.

As a final part of our study, we evaluated the proportion of reactions that each model contains from the other ones (Figure 10).

Since Recon3D is the most current and updated human metabolic model and encompasses all the other models being analyzed, it is the one who has a higher proportion in all the other models, with the exception of the HMR1. This model is the least integrated, even with its second version. This is mainly due to the dissimilarities of the nomenclature of both reactions and metabolites (even different from HMR2), which makes it harder to integrate.

When looking at the Recon family of models, Recon2 is almost entirely integrated with Recon1 and Recon3D has more than 80 % of the reactions on Recon2; however, Recon2 should have a higher proportion when compared to Recon3D as well as Recon3D when compared to Recon1. This can be explained, for example, as the removal or modification of certain metabolites or reactions throughout the iterations of the models that are not logged or due to errors on the integration process. In the former case, if a metabolite or reaction is altered in a next revision, the integration process may not recognize these changes and will lead to the creation of two clusters rather than a single one.

Although our results show that it is possible to integrate a large portion of most current human GSMMs, there are some limitations that need to be addressed as part of future work.

The fact that we impose that integrated reactions must contain exactly the same stoichiometry-metabolite pairs further impairs the algorithm’s ability to capture these minor differences between revisions and models as integrated entities. On the other hand, some metabolite clusters may wrongly contain several unrelated metabolites that, when used to integrate reactions, may lead to large reaction clusters that group reactions that are not identical. This may occur when different metabolites share the same information, grouping them as a cluster (as is the case for some cofactors). One solution would be restricting the amount of databases included in the integration, at the cost of losing potentially correct clusters due to the loss of information. Manual curation could also solve these problems although the purpose of this work is to implement an automated method.

## 4 Conclusions and Future Work

In the present work, we attempt to enrich human metabolic models with external database identifiers to improve one of their main limitations, namely, their integration with other biological information repositories. With the presented pipeline, we provide a semi-automated tool to enrich the metabolite information that each model contains. Using these results, we decided to integrate the reactions for all the models present in the most recent reconstruction of the human metabolism (Recon3D). Although this process shows some interesting results, issues that come from Gene to Protein Rules and reactions that include more than one compartment have to be addressed in future work.

Concluding, this work demonstrates that our presented pipeline can help to bridge the different human metabolic models. Despite requiring some manual curation, this work is a good starting point to bridge these models to achieve one of the most desired objectives in this field of study, the reconstruction of a unified human metabolic model.
